# An interpretation of the new international MAP guideline for the management of Milk Allergy in Primary Care

**DOI:** 10.1186/s13601-017-0171-x

**Published:** 2017-09-22

**Authors:** Paul Netts, Louise J. Michaelis

**Affiliations:** 1 Benfield Park Medical Group, Newcastle Upon Tyne, NE6 4QD UK; 2 Department of Paediatric Allergy, Great North Children’s Hospital, Newcastle Upon Tyne Hospitals NHS Foundation Trust, Newcastle Upon Tyne, NE1 4LP UK; 3 Institute of Cellular Medicine, University of Newcastle, Newcastle Upon Tyne, UK

**Keywords:** Cow’s milk allergy, Guideline, Primary care

## Abstract

General Practitioners suffer from guideline fatigue. They come fast and furious in many complicated forms. Cow’s milk allergy (CMA) is one of the most common presentations of food allergy seen in early childhood presenting to primary and secondary care. The early and accurate diagnosis continues to be highlighted in many countries worldwide. International surveys have found that primary care clinicians would like clearer explanations for the options for the diagnosis of CMA and in so doing a means to increase their understanding of management options for both IgE and Non IgE mediated CMA. In 2013 in response to General Practitioner demands, the UK guideline, ‘Diagnosis and management of non-IgE-mediated CMA in infancy—a UK primary care practical guide’ was published in this journal. This Milk Allergy in Primary Care (MAP) guideline outlines in simple algorithmic form how to diagnose, manage and refer children with CMA in a primary care setting. Based on the international uptake of the MAP guideline, a global practical guideline International MAP is presented by the Venter and Brown et al to help practitioners in primary care settings. It incorporates further published UK guidance, feedback from UK healthcare professionals and affected families and, importantly, also international guidance and expertise.

Primary care has guideline fatigue. The sheer number and rate with which they appear is challenging and currently General Practitioners (GPs) in the United Kingdom (UK) do not follow the vast majority of them at the best of times [1]. This is of particular concern as, in many respects, GPs and other community clinicians are increasingly bound by them in three important ways (a) clinically, where they offer an easy way of maintaining up-to-date care, (b) legally, where they often define acceptable practice, and (c) ethically, given their impact on practitioner autonomy, patient empowerment, standardisation of care and management of costs [2].

Given this current climate, how can a guideline about the management of IgE and Non IgE mediated cow’s milk allergy (CMA) in primary care in the UK begin to have an impact? After all, primary care, in the case of non-IgE CMA, is being asked to manage a condition that has no current diagnostic test, seems self-limiting and, through the eyes of the uninitiated, does not appear to result in detrimental effects on future health for children if left unrecognised and untreated. Add to this a lingering opposition to the very existence of non-IgE CMA as a clinical entity, both in primary and secondary care, and the challenge seems even greater. Yet despite all this, the original “*Milk Allergy in Primary Care (MAP)*” UK guideline [3], which was originally launched in 2013, has been well received and is being successfully implemented globally [4]. This is particularly evident when it is presented with a clear algorithm and a simple accompanying training package for both breast and formula fed infants.

For a guideline to produce such a change in behavior suggests that it is imparting new knowledge on those that read and use it [5]. This prior lack of knowledge in UK primary care, along with an associated lack of confidence in managing food allergy, had been highlighted years earlier [6]. These deficiencies manifested themselves with delays in diagnosis, multiple unnecessary appointments and significant inappropriate prescribing of formula milks. To further compound the situation there was limited knowledge about the types of formula milks available, a dearth of dietetic support as well as a striking disconnect between primary and secondary care with many patients turning to tertiary care for assistance.

The 2003 Royal College of Physicians (UK) report, “*Allergy the unmet need: a blueprint for better patient care*” called for the development of “*co*-*ordinated allergy services delivered seamlessly by primary and secondary care”.* It was envisaged that ultimately these services would be led by primary care so education and guidance was clearly required [7, 8]. Fortunately the evidence at the time was that the appetite for such training existed and that targeted education was effective in increasing confidence amongst GPs [9]. In 2010 the World Allergy Organization (WAO) *Diagnosis and Rationale for Action against Cow’s Milk Allergy (DRACMA) guideline* [10] further clarified the diagnosis, treatment and subsequent prognosis of CMA and gave advice for every presentation from suspicion to anaphylaxis.

In 2011 the National Institute of Health and Care Excellence (NICE) UK guidance [11] asserted that primary care needed a guideline to manage CMA but fell short of informing GPs what to do, let alone provide the treatment algorithm they sought. Two years later the MAP guideline was published which did exactly that [3]. It originated from separate infant feeding programmes in Northern Ireland before developing into a national UK guideline. It provided the first treatment algorithm and pathway of care for CMA with the focus on non-IgE CMA (given its potential for management solely in primary care). Included in this pathway was visual instruction that allowed for formal diagnosis of non-IgE CMA not previously offered by the UK NICE guidance.

To address a clinical need however is not enough by itself to ensure the quality of any guideline; this requires that the development process be robust by ensuring clarity, applicability, involvement of all stakeholders and a strong evidence base [8]. The MAP guideline met these criteria and, as 60,000 downloads (personal communication) worldwide will attest to, remains extremely popular despite a strong UK focus. The new iMAP guideline however, has identified that a more international approach is needed and has gained an “i” in recognition of this [12]. Whilst the UK has the highest prevalence of non-IgE CMA in Europe [13] and prescribes more amino acid formula milk as first choice than anywhere else in Europe the problem is certainly not confined to British shores [14]. Changes have therefore been made that are sensitive to the needs of other countries.

The general layout of the new iMAP guideline and the focus on non-IgE CMA remains unchanged; however the alterations that have been made aim to improve the ease and efficacy of everyday food allergy management. One of the ways in which it seeks to do this is by aiding the process of making an accurate and timely diagnosis [11]. The importance of the early recognition of food allergy in the first 1000 days of a child’s life has been shown to be crucial [15]. Emphasis is now placed on the need for an appropriate allergy focussed history and physical examination by a clinician at first contact. In the non-IgE section there is a more detailed list of possible presenting symptoms. Clearer instruction, in the form of a written protocol, for both parent and health care professional alike, outlines the withdrawal and subsequent home re-introduction of cow’s milk that is necessary to confirm or refute the diagnosis. Further helpful documentation is provided: an initial factsheet for parents, an amended less intense milk ladder and a sheet of useful recipes.

The iMAP guideline further supports the vital role of the community dietitian in every child with a food allergy. Dietitians in the UK bring their own expertise to the platform; promoting the importance of breast feeding, monitoring of growth, supervising vitamin and calcium supplementation for both mother and child, maintaining an understanding of the different types of formula milks and providing a gateway to food inclusion rather than exclusion. Home re-introduction (double blind placebo controlled in the Netherlands guideline) is vital if CMA is refuted and the dietitian is crucial in supporting this outcome.

The challenge remains however to ensure that primary care is educated enough to recognise that an array of symptoms and conditions, that can stand alone as clinical entities in and of themselves, may in fact be the presentation of allergy. A survey in 2015 demonstrated that only a third of GPs felt equipped to deal with food allergy [16]. It is essential that clinicians are aware that appropriate early treatment could halt or minimise the likelihood of these children developing other co-morbid allergic conditions such as atopic dermatitis, asthma and allergic rhinitis [17]. Such quality management could save everyone time and resources [6] as well as potentially reducing the incidence and prevalence of these increasingly burdensome conditions. There is a clinical imperative to manage in this way as in the UK alone atopic disease has trebled in the last 20 years, with 13 million people below their mid-forties having two or more allergies [7].

In conclusion, the iMAP Guideline, is an evidence based, practical clinical algorithm that will further enhance the ability of health care professionals in primary care to better recognise and manage CMA. They can be used as the foundation for the training and education that will ultimately result in a primary care that is more confident and better placed to receive and implement the next advances in allergy management. The use of probiotics in breast fed babies, synbiotics in formula milks [18–20], food desensitisation [21] and the induction of oral tolerance will represent the next management challenges for primary care. All this makes seamless collaboration with secondary and tertiary services, along with strong dietetic and strong community nursing community support (e.g. in the UK health visitor support), all the more important. Improved patient care and safety, with a reduction in overall health costs be they social, emotional and/or financial, will be evident. By sowing the seeds of early recognition and diagnosis, and following this with evidence based management, patients and clinicians alike will reap the benefits both now and in the future. The iMAP guideline will play no small part in contributing to this.

## Abbreviations

CMA: cow’s milk allergy
GP: General Practitioner
iMAP: International Milk Allergy in Primary Care
MAP: Milk Allergy in Primary Care
NICE: National Institute of Health and Care Excellence
WAO: World Allergy Organization
